# Low Field MRI Measurements of the Normal Canine Trigeminal Nerve

**DOI:** 10.3389/fvets.2020.00274

**Published:** 2020-05-19

**Authors:** Charlotte E. Swain, Giunio B. Cherubini, Panagiotis Mantis

**Affiliations:** Dick White Referrals, Six Mile Bottom, Cambridgeshire, United Kingdom

**Keywords:** reference range, idiopathic trigeminal neuropathy, dog, magnetic resonance imaging, contrast-enhanced, multiplanar reconstruction (MPR)

## Abstract

There is no available measuring protocol and reference range for the normal canine trigeminal nerve. This can be problematic in cases of suspected bilateral trigeminal neuropathy since contralateral nerves cannot be a usefully compared. Trigeminal nerves and brain measurements were retrospectively assessed via multiplanar reconstruction (MPR) of 3DT1 post-contrast MR sequences from 137 dogs with no signs or diagnosis of trigeminal disease. Direct measurements of vertical brain height (BH), trigeminal nerves transverse height (TTH) and trigeminal nerves width in dorsal reconstruction (TDW) were made in a plane immediately caudal to the foramen ovale and used to derive trigeminal nerve-to-brain (NB) ratios, including height-to-brain ratio (HBR) and width-to-brain ratio (WBR). HBR (0.09, IQR = 0.08-0.09) and WBR (0.10, IQR = 0.09-0.11) maintained more consistent values across the study population compared to direct measurements of TTH (3.72, IQR = 3.42-4.07) and TDW (4.35 +/− 0.63). Calculated normal reference intervals for HBR and WBR were 0.07-0.11 and 0.08-0.13, respectively and the largest NB ratios recorded in normal dogs were 0.13 and 0.14 for HBR and WBR, respectively. All measurements varied proportionally with weight, including HBR (*r* = 0.41, *p* < 0.0001) and small dogs had a significantly smaller HBRs compared to medium (*p* = 0.0294), large (*p* < 0.0049) and giant dogs (*p* < *0.0044*). Median HBR was the same across skull types (0.09), however *post-hoc* analysis detected significantly smaller HBRs in brachycephalic compared to mesaticephalic dogs (*p* = 0.0494). In conclusion, trigeminal NB ratios may allow for accurate, objective assessment of the canine trigeminal nerves on MRI but further quantification of the effects of weight and skull type on suggested reference intervals is needed.

## Introduction

The trigeminal nerve is the fifth and largest cranial nerve; it has three main branches, the ophthalmic and maxillary nerves, that are purely sensory and innervate the face, mouth, eye and nasal cavity, and the mandibular nerve, a mixed motor and sensory branch, which provides somatic motor innervation to the muscles of mastication (1). The origin, course and foramina of the normal canine trigeminal nerve have been described in one anatomical study using magnetic resonance imaging (MRI) in dogs (2), however no normal range of nerve measurements exists in the veterinary literature.

The trigeminal nerves emerge from the brainstem at the junction of the caudal mesencephalon and pons and are most readily identified just caudal to the interthalamic adhesion, where they are orientated horizontally and course laterally over the lateral projections of the basisphenoid bone (2, 3). Immediately cranial—at the level of the interthalamic adhesion—the largest branch (the mandibular nerve) exits the skull via the foramen ovale (2). The remaining smaller branches (the maxillary nerve and the ophthalmic nerve) have longer intracranial courses and exit the skull via the foramen rotundum and the orbital fissure, respectively (2). Mild post-contrast enhancement of intracranial trigeminal structures is reported to occur in normal dogs and humans without signs of trigeminal disease (2, 4, 5). In dogs specifically, T1-weighted (T1W) post-contrast enhancement of the trigeminal ganglion occurs in up to 100% of unaffected animals and the majority of dogs also show normal post-contrast enhancement of the entire intracranial trigeminal nerve and brainstem nuclei (4).

Dogs present more commonly with bilateral trigeminal disease than unilateral neuropathy (6). However, diagnostic MRI is more frequently undertaken in the latter presentation because the contralateral nerve can serve as a normal control for comparing size, signal intensity, and degree and pattern of contrast enhancement (7–11). In cases of bilateral trigeminal neuropathy, contralateral nerves cannot be usefully compared so a presumptive diagnosis of idiopathic trigeminal neuropathy (ITN) is often made on the basis of resolving or non-progressive clinical signs (12, 13). Only one report has described MRI findings in a case of bilateral ITN in a dog, in which both trigeminal nerves and ganglia revealed mild post-contrast enhancement similar to unaffected animals (4, 14).

The available studies reporting the origin, course and post-contrast enhancement of the normal canine trigeminal nerve utilized standard 2D spin echo sequences acquired on high field magnets (2, 4). Achieving similar resolution spin echo sequences with low field systems—as in the current study—would result in significant time penalties and prohibitively long acquisition times (15). Therefore, on low field systems, when high resolution images are required (i.e., to visualize cranial nerves), 3D volumetric sequences are acquired. 3D datasets benefit from the absence of an interslice interval, increased signal-to-noise ratio—so slices can be thinner—and the ability to select for isotropic voxels, all of which increase spatial resolution and allow images to be reformatted into different planes using multiplanar reconstruction (MPR) (16). Long acquisition times are also offset in 3D scans with the use of short repetition time (TR) gradient echo pulse sequences and the possibility to perform MPRs (16). It is clear from the literature that relative enlargement of the trigeminal nerve with respect to the contralateral nerve is only useful for diagnosing unilateral trigeminal pathology in dogs (7–11) and contrast enhancement alone does not reliably differentiate normal from abnormal trigeminal nerves (4). In spite of this, no standardized measuring protocol or published normal range exists for the canine trigeminal nerve on MRI.

The purpose of this study was to establish an objective, normalized measuring protocol for the canine trigeminal nerve on MRI and to record the effects of weight, sex and skull type on measured values.

## Materials and Methods

A single center, retrospective, observational study was performed. Electronic patient records were searched to identify cases, for which high resolution, three-dimensional T1-weighted post-gadolinium^1^ (3DT1+C) sequences had been acquired during brain MRI scans. 3DT1+C sequences were obtained using the following parameters (TR = 20.2 ms; TE = 10.3 ms; FA = 10^o^; ST = 1.0 mm; FOV = 200 mm and matrix = 512 × 512) on an 0.4T magnet^2^. All dogs were anesthetized for scanning with IV propofol^3^ and maintained on isoflurane^4^ in oxygen. Age, sex, neuter status, weight, breed, presenting clinical signs and imaging diagnosis were recorded for all cases. Dogs with clinical signs suggestive of trigeminal nerve pathology or subjective trigeminal nerve enlargement on imaging were automatically excluded from the study. Cases found to have multifocal CNS lesions, altered brain anatomy, inflammatory CSF or contrast enhancement in cranial nerves in close proximity to the trigeminal nerve (facial and vestibulocochlear nerves) were also excluded. All cases were reviewed by a board-certified neurologist (GBC) to exclude any possible trigeminal nerve involvement.

**Figure 1:**
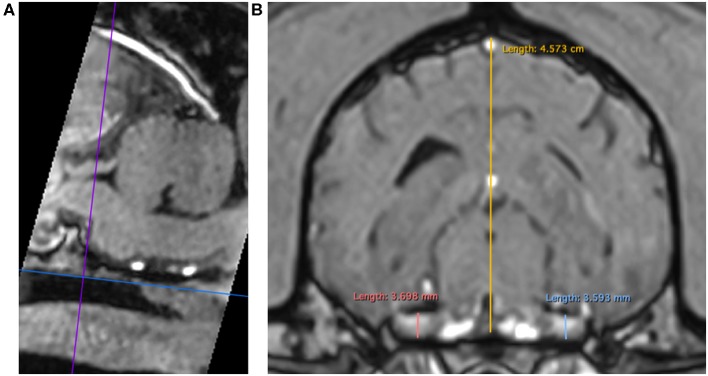
MPR of a 3DT1+C sequence of the brain of a Labrador Retriever (TR = 20.2 ms, TE = 10.3 ms, ST = 1 mm). Saggital reconstruction **(A)**. The dorsal axis (blue line) is adjusted parallel to the basisphenoid bone to ensure the transverse axis (purple line) is perpendicular. Transverse reconstruction **(B)**. Trigeminal nerves are identified coursing horizontally just caudal to the foramen ovale. Caliper measurements for HBR calculation: TTH left (light blue line), TTH right (coral pink line), and BH (yellow line). MPR, multiplanar reconstruction; 3DT1+C, three-dimensional T1-weighted post-contrast; TR, repetition time; TE, echo time; ST, slice thickness; TTH, trigeminal nerve transverse height; BH, brain height; HBR, height-to-brain ratio.

**Figure 2:**
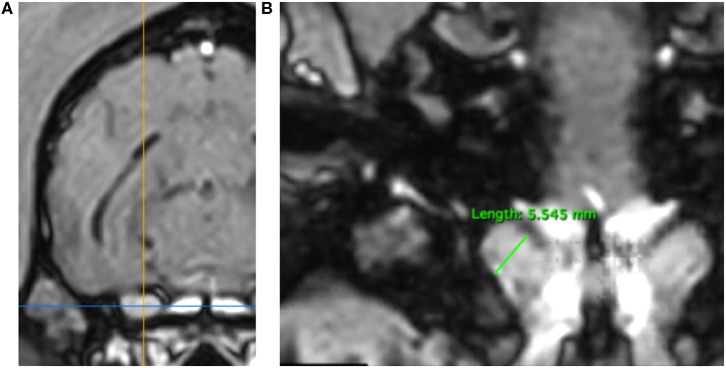
MPR of a 3DT1+C sequence of the brain of a Springer Spaniel (TR = 20.2 ms, TE = 10.3 ms, ST = 1 mm). Transverse reconstruction **(A)**. The intersection of the saggital axis (yellow line) and dorsal axis (blue line) is centered over each trigeminal nerve in turn. Dorsal reconstruction **(B)**. The trigeminal nerve is identified coursing craniolaterally over the wings of the basisphenoid bone. Caliper measurement of TDW (green line) for calculation of WBR. TDW, trigeminal nerve dorsal width; WBR, width-to-brain ratio.

3DT1+C brain sequences were reformatted using the multiplanar reconstruction (MPR) tool on Horos medical image viewer software^5^ into sagittal, transverse and dorsal planes. The dorsal axis was rotated to create an arbitrary dorsal plane parallel to the basisphenoid bone in order to orientate transverse reconstructions perpendicular to the sphenoid bone and match the transverse orientation of conventional 2D T1W spin echo sequences (17). At this location, caliper measurements were made of vertical brain height (BH) and vertical height of both left and right trigeminal nerves, denoted trigeminal nerves transverse height (TTH) (Figures 1A,B). The intersection of the sagittal and dorsal axes was then centered over the left and right trigeminal nerves, in turn, and the maximal widths of the nerves in dorsal reconstruction, trigeminal nerves dorsal width (TDW), was recorded (Figures 2A,B). Each measurement was performed three times by a single observer (CES) and the highest value was recorded. Nerve-to-brain (NB) ratios were then calculated by dividing TTH and TDW measurements by BH measurements to yield trigeminal nerve height-to-brain ratios (HBRs) and width-to-brain ratios (WBRs), respectively. Finally, left and right NB ratios were averaged to produce single HBR and WBR values for each patient.

All statistical analyses were performed on GraphPad Prism 7 Software^6^. Variables were evaluated for Gaussian distribution via Shapiro-Wilk normality tests, following which appropriate statistical tests were performed to a standard significance level of *p* < *0.05*. Spearman's rank-order correlation coefficient was calculated to test the association between weight and outcome variables, including BH, TTH, TDW, HBR, and WBR and relationships were graphically represented through linear regression on scatter plots. Dogs were classified into small (<10 kg), medium (10- <25 kg), large (25- <40 kg), and giant (≥ 40 kg) weight groups and differences in mean TTH and TDW were assessed with ordinary one-way analysis of variance (ANOVA) and Tukey's multiple comparisons. NB ratios were nonparametrically distributed within weight groups and differences in median values were assessed via the Kruskal-Wallis statistic and Dunn's *post-hoc* multiple comparisons. Differences in median NB ratios were similarly assessed with respect to skull type *(brachycephalic, mesaticephalic and dolichocephalic*) and a Mann-Whitney *U*-test was performed to compare median NB ratios between male and female dogs.

Reference intervals (RIs) may be estimated from skewed datasets by log-transforming data to conform to normality prior to performing RI calculations, followed by antiloging calculated limits back to linear scale (18). RIs were, therefore, calculated for skewed datasets (TTH, HBR and WBR) using to the following equation, where y = the outcome variable, y^1^ = log(y), SD y^1^ = the standard deviation of y^1^, L1 = lower limit of y^1^, U^1^ = upper limit of y^1^, L = lower limit of y in linear scale and U = upper limit of y in linear scale.

$$\begin{array}{lll} Reference\;interval\;y^{1} & = & mean\;y^{1}\pm 1.96\times SD\;y^{1}\\ L^{1} & = & mean\;y^{1}-1.96\;\times SDy^{1}\\ U^{1} & = & mean\;y^{1}+1.96\;\times SDy^{1}\\ L & = & 10^{L^{1}}\\ U & = & 10^{U^{1}} \end{array}$$

## Results

A total of 137 dogs met the inclusion criteria for the study; 74 male and 63 female dogs of bodyweights ranging from 1.6 to 96 kg and positively skewed toward a median of 14.8 kg. From the 137 dogs included in the study, 117 dogs were purebred with the Cavalier King Charles Spaniel (*n* = 14), Labrador Retriever (*n* = 12), Springer Spaniel (*n* = 10), Cocker Spaniel (*n* = 10), Boxer (*n* = 9), French Bulldog (*n* = 8), and Border Collie (*n* = 7) overrepresented. A further 20 dogs were classified as hybrids or uncategorized crossbreeds. The mean age of dogs in the study was eight years with a range of ten months to fifteen years. Forty-four dogs (32%) were classified as brachycephalic, 72 dogs (53%) were mesaticephalic and 15 dogs (11%) were dolichocephalic. Six dogs (4%) were uncategorized crossbreeds and could not be classified with respect to their skull type. MRI of the brain was unremarkable in 117 (85.4%) of study participants, including the following normal variants: caudal occipital malformation syndrome (8.8%, *n* = 12), senile cerebral atrophy (9.5%, *n* = 13) and mild cerebellar atrophy (0.7%, *n* = 1). Abnormal (non-trigeminal nerve) findings were present on MRI in 14.6% of dogs (*n* = 20), including chronic otitis media/interna (*n* = 8), infarction of the midbrain (*n* = 3) or thalamus (*n* = 1), focal contrast enhancement of the cerebellum (*n* = 1), thalamus (*n* = 1), frontal lobe (*n* = 1) or optic nerve (*n* = 1), leukariosis (*n* = 2), multifocal microbleeds (*n* = 1), and unilateral myositis secondary to a stick injury (*n* = 1).

**Figure 3:**
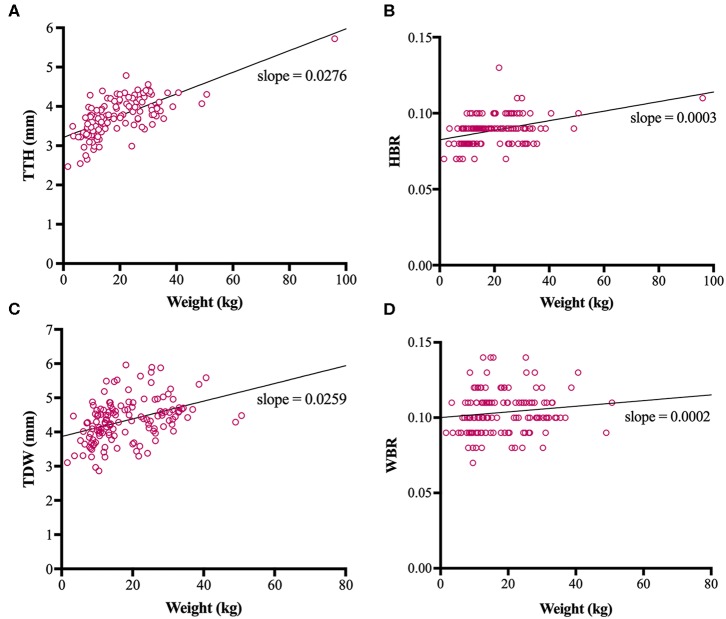
Scatterplots, linear regression lines of best fit and linear regression slopes for trigeminal nerves transverse height (TTH) vs. weight **(A)**, trigeminal nerves height-to-brain ratio (HBR) vs. weight **(B)**, trigeminal nerves dorsal width (TDW) vs. weight **(C)** and trigeminal nerves width-to-brain ratio (WBR) vs. weight **(D)**.

Direct trigeminal nerve height and width measurements, TTH and TDW, had average values of 3.72mm (IQR: 3.42-4.07) and 4.35 mm (+/− 0.63), respectively. Calculated NB ratios showed less overall variation than direct trigeminal nerve measurements with a median HBR of 0.09 (IQR: 0.08-0.09) and a median WBR of 0.10 (IQR: 0.09-0.11). Calculated RIs for direct trigeminal nerve measurements were 2.88-4.76 mm for TTH and 3.23-5.72 mm for TDW and for NB ratios were 0.07–0.11 and 0.08-0.13 for HBR and WBR, respectively. All outcome variables showed statistically significant positive correlation with weight, however, NB ratios had weaker correlation with weight than their directly measured counterparts (Table 1, Figure 3). A strong positive correlation was identified between weight and TTH (*r* = 0.68; *p* < *0.0001*) compared to only moderate positive correlation between weight and HBR (*r* = 0.41; *p* < 0.0001). Similarly, TDW was moderately correlated with weight (*r* = 0.46; *p* < 0.0001) whereas WBR was only weakly correlated (*r* = 0.19; *p* = 0.0245). Furthermore, regression line analysis showed that NB ratios increased less as a function of weight than direct measurements; the regression slopes of TTH and TDW vs. weight were 0.0276 and 0.0259 mm/kg, respectively, compared to regression slopes of only 0.0003 and 0.0002 /kg for HBR and WBR, respectively (Table 1, Figure 3).

**Table 1:** Linear regression analysis and Spearman's rank correlation for direct trigeminal nerve measurements, brain height and calculated nerve-to-brain (NB) ratios.

**Weight vs**.	**Regression slope**	**Regression slope 95% CI**	**Spearman's rank co-efficient (Rs)**	**Rs significance**
				**(*P* value)**
Trigeminal nerves transverse height (TTH)	0.0276 (mm/kg)	0.0226–0.0326 (mm/kg)	0.68	<0.0001
Trigeminal nerves dorsal width (TDW)	0.0259 (mm/kg)	0.0158–0.0359 (mm/kg)	0.46	<0.0001
Brain height (BH)	0.1498 (mm/kg)	0.1045–0.1950 (mm/kg)	0.50	<0.0001
Trigeminal nerves height-to-brain ratio (HBR)	0.0003 (1/kg)	0.0002–0.0004 (1/kg)	0.41	<0.0001
Trigeminal nerves width-to-brain ratio (WBR)	0.0002 (1/kg)	0.0001–0.0004 (1/kg)	0.19	0.0245

**Figure 4:**
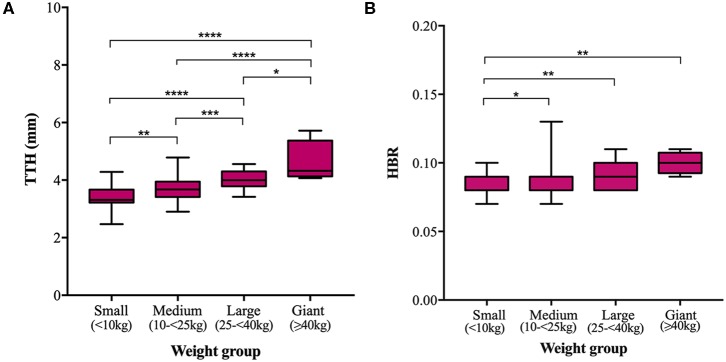
Box and whisker plots of trigeminal nerves transverse height (TTH) vs. weight groups **(A)** and trigeminal nerves height-to-brain ratio (HBR) vs. weight groups **(B)**.

**Figure 5:**
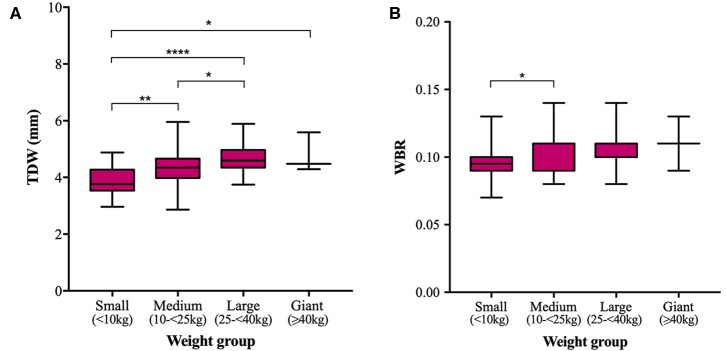
Box and whisker plots of trigeminal nerves dorsal width (TDW) vs. weight groups **(A)** and trigeminal nerves width-to-brain ratio (WBR) vs. weight groups **(B)**.

Mean TTH was 3.35, 3.68, 4.03, and 4.61 mm and mean TDW was 3.88, 4.34, 4.69, and 4.79 mm in small, medium, large, and giant dogs, respectively. Both TTH and TDW showed statistically significant increases with increasing weight group (*p* < 0.0001 and *p* < 0.0001, respectively), which existed between all pairs of groups for TTH (Figure 4A) and all pairs of groups for TDW except medium vs. giant and large vs. giant dogs (*p* = 0.5372 and *p* = 0.9908, respectively) (Figure 5A). Median HBRs were similar between weight groups: 0.08, 0.09, 0.09 and 0.10 for small, medium, large, and giant dogs, respectively. *Post hoc* comparisons revealed significantly smaller HBRs in small dogs compared to medium (*p* = 0.0294), large (*p* = 0.0049), and giant dogs (*p* = 0.0044) (Figure 4B); no differences in HBR were observed between medium vs. large (*p* > 0.9999), medium vs. giant (*p* = 0.1345) or large vs. giant (*p* = 0.4091) dogs. Median WBRs varied even less between weight groups: 0.10, 0.11, 0.10, and 0.11 for small, medium, large and giant dogs, respectively and the only difference to reach statistical significance was a smaller WBR in small vs. medium dogs (*p* = 0.028) (Figure 5B).

**Figure 6:**
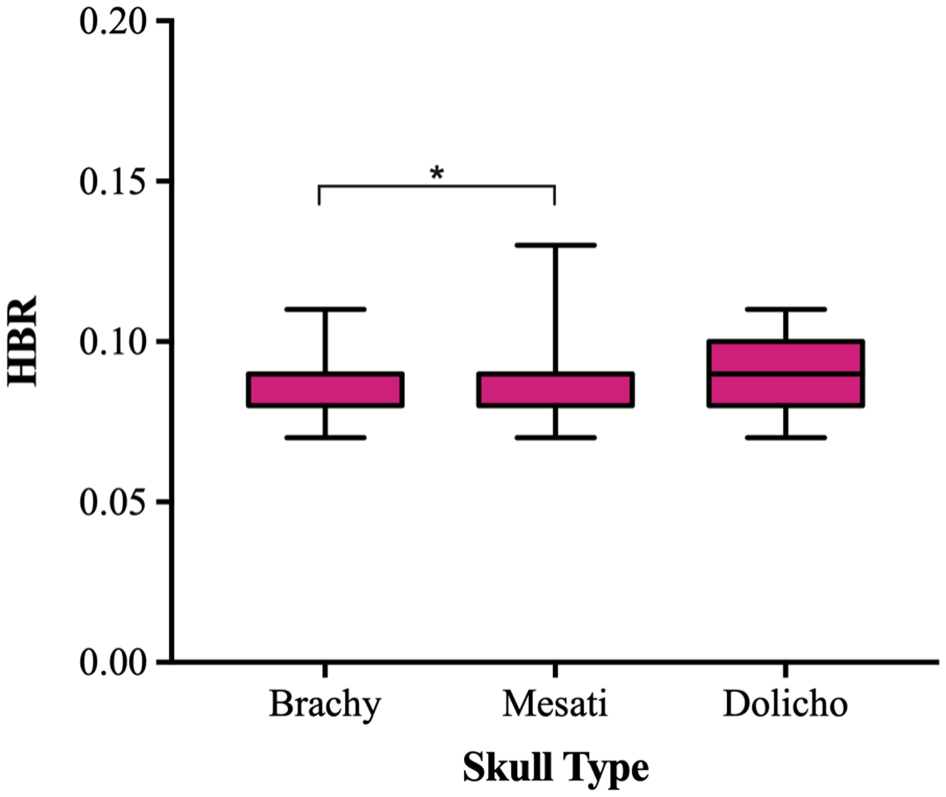
Box and whisker plot of trigeminal nerves height-to-brain ratio (HBR) vs. skull type.

Median HBR was the same (0.09) for all three skull types, however datasets were non-normally distributed and brachycephalic dogs had significantly smaller HBRs overall compared to mesaticephalic dogs (*p* = 0.0494) (Figure 6); no difference was detected in HBRs between brachycephalic and dolichocephalic dogs or mesaticephalic and dolichocephalic dogs (*p* = 0.424 and *p* > 0.999, respectively). Median WBR was 0.10, 0.10, and 0.11 in brachycephalic, mesaticephalic and dolichocephalic dogs, respectively. There were no statistically significant differences detected in WBR on the basis of skull type (*p* = 0.0924).

In both male and female dogs, median HBR was 0.09 and median WBR was 0.10. There was no statistically significant difference detected in either NB ratio on the basis of sex (*p* = 0.0657 and *p* = 0.6603 for HBR and WBR, respectively).

## Discussion

To the authors' knowledge, this is the first report outlining normal canine trigeminal nerve measurements on MRI and the first study to calculate and compare trigeminal nerve neural ratios. Neural ratios help to normalize measurements within a population when absolute values vary significantly. In human medicine, cranial nerve neural ratios are increasingly quoted as reference intervals (RIs) and disease markers in MR studies; for example, optic nerve-to-optic tract ratios provide better assessment of pediatric optic pathways than absolute diameters because they do not vary with age (19), a larger facial nerve-to-facial canal ratio conveys increased risk of Bell's Palsy (20, 21) and a reduced cochlear nerve-to-facial nerve ratio is associated with sensorineural hearing loss (22).

Unfortunately, no direct comparison can be drawn between human and canine trigeminal diseases because pure trigeminal motor neuropathy, the most common disease presentation in dogs, is reportedly rare in people (23). However, trigeminal nerve measurements, including cross-sectional area and total nerve volume are reported as objective measures of trigeminal atrophy in patients with trigeminal neuralgia and both correlate with disease morbidity and prognosis (24–26). There is also new evidence to support calculation of neural ratios in canine subjects as a means to control for variation in cranial morphology; Noh et al. calculated interthalamic adhesion thickness-to-brain height ratios in a study assessing cognitive dysfunction in dogs and found that calculated ratios controlled for wide variations in brain height and skull type and did not vary as a function of bodyweight or gender (27).

In the present study, trigeminal NB ratios exhibited less data spread (IQRs and ranges) in the studied population than direct trigeminal nerve measurements and showed weaker correlation and gentler regression line slopes with respect to weight than corresponding direct measurements. Direct nerve measurements also showed more significant differences between weight groups; TTH was significantly different between all pairs of weight groups and TDW was significantly different between all groups except medium vs. giant and large vs. giant dogs. Failure to detect differences between groups could reflect a type 2 error and is particularly likely for comparisons drawn against giant dogs because the sample population was small. All significant differences in HBR and WBR were detected between small dogs and dogs of other weight groups. No differences in either HBR or WBR were detected between medium and large, medium and giant or large and giant dogs. Overall, both direct measurements and NB ratios showed little variation within the study population. Therefore, a larger sample size might be required to prevent underpowering in future studies.

A recent paper found that for any given external skull type, as measured by the cephalic index (ratio of cranial width to cranial length), smaller breed dogs have significantly higher neurocephalic indices (ratio of brain width to brain length) and thus more spherical brains than larger breed dogs (28). This difference in brain morphology in smaller-bodied dogs may explain why small dogs in our study had significantly smaller NB ratios compared to dogs with more ovoid brains in heavier weight groups. In addition, brachycephalic dogs had significantly smaller HBRs than mesaticephalic dogs, which might be explained by brachycephalic breed-related attenuation of rostro-caudal skull growth and resultant compensatory increase in height of the cranial vault and brain (29, 30). Regardless whether differences between groups reached statistical significance, the overall magnitude of differences was very small and is unlikely to have real-world implications, especially given person-to-person variability and errors implicit to measuring small structures on MRI (31).

Suggested RIs for trigeminal NB ratios were calculated via logarithmic data transformation (27) and are given as follows: 0.07–0.11 for HBR and 0.08–0.13 for WBR. Whilst these RIs, provide useful information regarding the normal size of the canine trigeminal nerve on MRI, the parametric statistical methods used to calculate these limits can result in excessively narrowed confidence intervals and strict ranges (32). The risk of RI calculations producing overly conservative limits in the present study, is made more likely given that originally skewed datasets required log-transformation to meet requirements for RI calculations. To that end, the largest NB ratios recorded in this population of normal dogs were 0.13 and 0.14 for HBR and WBR, respectively, and therefore, these values could also be quoted as less conservative maximum limits.

Although the low field 0.4T magnet used in this study necessitated the use of high resolution 3DT1+C sequences to accurately identify the trigeminal nerves, careful use of MPR tools ensured that transverse reconstructions matched the typical transverse orientation used by most institutions for conventional T1W spin echo sequences (26). In this standard orientation, perpendicular to the hard palate and sphenoid bone, the trigeminal nerves are viewed parallel and appear horizontal (3), which facilitates ease of measuring. Therefore, institutions with higher spatial resolution magnets (1.5T or 3T) will be able to acquire trigeminal nerve height measurements and neural ratios directly comparable to our study from conventional T1W post-contrast sequences without the need for volumetric datasets. Comparable trigeminal nerve width measurements and neural ratios might also be possible on conventional dorsal T1W post-contrast sequences on high-field MRI providing dorsal alignment is parallel to the hard palate; this is the most common dorsal orientation used by institutions; however, some prefer to align perpendicular to the long axis of the hippocampus as advised by epilepsy-specific protocols (33). The latter is not recommended for trigeminal nerve measurements as inclinations of the dorsal axis with respect to the basisphenoid bone, displays the trigeminal nerves with varying degrees of obliquity, which may lead to inaccuracies of measuring or non-comparable results.

A requirement for volumetric sequences to perform nerve measurements in this study limited the potential sample population as 3D datasets are non-standard in brain MRI protocols. One observer also made all trigeminal nerve and brain measurements and, therefore, the influence of interobserver variability on direct measurements and calculated NB ratios is unknown, though the fact that the largest of three measurements was recorded as the correct potentially limits this possible variability.

In spite of these limitations, this study provides the first objective, measuring protocol and provisional RIs for the normal canine trigeminal nerve on MRI. Recommended normal ranges for direct measurements are 2.88–4.76 and 3.23–5.72 mm for TTH and TDW, respectively and for calculated NB ratios are 0.07–0.11 and 0.08–0.13 for HBR and WBR, respectively. NB ratios vary less with respect to weight than direct measurements and may allow for better comparisons between dogs. Follow-up studies should aim to further quantify the effects of weight and skull type on NB ratios, evaluate interobserver agreement and assess NB ratios in confirmed cases of trigeminal neuritis and idiopathic neuropathy.

## Data Availability Statement

The datasets generated for this study are available on request to the corresponding author.

## Ethics Statement

This study was carried out in accordance with the principles of the Basel Declaration and recommendations of the ESRC guidelines, Research Ethics Committee, University of Nottingham. The protocol was approved by the Ethics Clinical Review Panel, School of Veterinary Medicine, University of Nottingham, Sutton Bonington Campus. Specific consent to use animal clinical data was not required as pet owners gave written informed consent to retain their animal's clinical information for use in future clinical studies on hospital admission.

## Author Contributions

PM conceived and supervised the study. GC advised on case inclusion. CS performed the data collection, analytic calculations, and wrote the first draft of the manuscript. All authors were involved in the editing of the manuscript.

## Conflict of Interest

The authors declare that the research was conducted in the absence of any commercial or financial relationships that could be construed as a potential conflict of interest.

## Footnotes

^1^Gadoteric acid (Dotarem), Guerbet, Roissy CdG Cedex, France.

^2^0.4 Tesla Aperto Lucent magnet, Hitachi Medical Corporation, Tokyo, Japan.

^3^Propoflo Plus, Zoetis UK Limited, London, UK.

^4^Isoflo, Zoetis UK Limited, London, UK.

^5^Horos free open source software (FOSS), Version 3.0, sponsored by Nimble Co LLC Purview, Annapolis, MD, USA.

^6^GraphPad Prism statistical software, Version 7.0a, GraphPad Software, San Diego, CA, USA.
